# Cell senescence-associated genes predict the malignant characteristics of glioblastoma

**DOI:** 10.1186/s12935-022-02834-1

**Published:** 2022-12-16

**Authors:** Chenyang Tan, Yan Wei, Xuan Ding, Chao Han, Zhongzheng Sun, Chengwei Wang

**Affiliations:** 1 Department of Neurosurgery, The Second Hospital of Shandong University, Jinan, Shandong People’s Republic of China; 2 Department of Neurology, The Second Hospital of Shandong University, Jinan, Shandong People’s Republic of China

**Keywords:** Glioblastoma, Cell senescence-associated genes, Tumor microenvironment, Pan-cancer analysis, Drug sensitivity

## Abstract

**Background:**

Glioblastoma (GBM) is the most malignant, aggressive and recurrent primary brain tumor. Cell senescence can cause irreversible cessation of cell division in normally proliferating cells. According to studies, senescence is a primary anti-tumor mechanism that may be seen in a variety of tumor types. It halts the growth and spread of tumors. Tumor suppressive functions held by cellular senescence provide new directions and pathways to promote cancer therapy.

**Methods:**

We comprehensively analyzed the cell senescence-associated genes expression patterns. The potential molecular subtypes were acquired based on unsupervised cluster analysis. The tumor immune microenvironment (TME) variations, immune cell infiltration, and stemness index between 3 subtypes were analyzed. To identify genes linked with GBM prognosis and build a risk score model, we used weighted gene co-expression network analysis (WGCNA), univariate Cox regression, Least absolute shrinkage and selection operator regression (LASSO), and multivariate Cox regression analysis. And the correlation between risk scores and clinical traits, TME, GBM subtypes, as well as immunotherapy responses were estimated. Immunohistochemistry (IHC) and cellular experiments were performed to evaluate the expression and function of representative genes. Then the 2 risk scoring models were constructed based on the same method of calculation whose samples were acquired from the CGGA dataset and TCGA datasets to verify the rationality and the reliability of the risk scoring model. Finally, we conducted a pan-cancer analysis of the risk score, assessed drug sensitivity based on risk scores, and analyzed the pathways of sensitive drug action.

**Results:**

The 3 potential molecular subtypes were acquired based on cell senescence-associated genes expression. The Log-rank test showed the difference in GBM patient survival between 3 potential molecular subtypes (P = 0.0027). Then, 11 cell senescence-associated genes were obtained to construct a risk-scoring model, which was systematically randomized to distinguish the train set (n = 293) and the test set (n = 292). The Kaplan-Meier (K-M) analyses indicated that the high-risk score in the train set (*P* < 0.0001), as well as the test set (P = 0.0053), corresponded with poorer survival. In addition, the high-risk score group showed a poor response to immunotherapy. The reliability and credibility of the risk scoring model were confirmed according to the CGGA dataset, TCGA datasets, and Pan-cancer analysis. According to drug sensitivity analysis, it was discovered that LJI308, a potent selective inhibitor of RSK pathways, has the highest drug sensitivity. Moreover, the GBM patients with higher risk scores may potentially be more beneficial from drugs that target cell cycle, mitosis, microtubule, DNA replication and apoptosis regulation signaling.

**Conclusion:**

We identified potential associations between clinical characteristics, TME, stemness, subtypes, and immunotherapy, and we clarified the therapeutic usefulness of cell senescence-associated genes.

**Supplementary Information:**

The online version contains supplementary material available at 10.1186/s12935-022-02834-1.

## Introduction

Glioblastoma (GBM), known as a grade IV glioma, is one of the most common primary cancers of the brain [1]. Despite surgical resection and adjuvant therapy, the median overall survival of GBM patients is only about 12–15 months, and the 5 year survival rate is well below 10% [2, 3]. The highly aggressive nature and the high recurrence rate of GBM increase the difficulty and failure rate of treatment to a large extent. Therefore, it is extremely significant to explore new treatment methods and treatment modalities.

Cell senescence can be defined as the permanent, irreversible cessation of the normal proliferating cell cycle [4]. Senescence’s pleiotropic character has been linked to good effects on a variety of biological processes, including immunological clearance, wound healing, and embryogenesis, as well as a reduction in mutation accumulation and eventual cancer [5, 6]. Senescent cells are cumulative with age in normal tissues and have been associated with degeneration and senescence of whole organisms. Mechanistically, the expression of pro-senescence functions is associated with the limitation of the regenerative capacity of stem and progenitor cells as well as the secretion of bioactive molecules (the so-called SASP), specifically pro-inflammatory and matrix-modifying peptides [7, 8]. At present, many experts and scholars believe that mitochondrial dysfunction is another major way to affect cell senescence. Senescence-associated mitochondrial dysfunction (SAMD) is closely related to the continuous DNA damage response signal or the expression and accumulation of genes related to cell senescence [9, 10]. The expression of cell senescence-associated genes is highly conserved in different tissues and organs, even in different species. At present, the research on cell senescence-associated genes mainly focuses on prolonging life spans and promoting health. There are other relevant studies on the function and role of cell senescence-associated genes in various malignancies, especially the function and role of cell senescence-associated genes in tumor infiltration and recurrence, but there are a relatively small number of these.

Immunotherapy normalizes the antitumor immune response that attacks cancer cells and more significant gains in cancer-related treatments [11]. Under normal circumstances, Immune cells in the tumor microenvironment (TME) have ability to recognize and eradicate tumor cells [12]. However, cancer cells can harmonize the host immune system to evade immune surveillance by tumor-induced immunosuppressive effect, antigenic modulation, tumor-induced generation of exempted areas and downregulating of tumor immunogenicity [13, 14]. In clinical applications, immunotherapy has achieved remarkable results in the treatment of certain tumors. For example, pembrolizumab resulted in significantly longer overall survival for patients with locally advanced or metastatic non-small cell lung cancer compared to chemotherapy [15]. However, there are still multiple challenges with cancer immunotherapy [16, 17]. According to statistics, only 13% of patients with malignancies respond to immunotherapy, Patients with a significant clinical response may eventually experience cancer progression after several years due to factors such as acquired drug resistance [18, 19]. Moreover, immune-related adverse events (irAEs) continue to occur at a significant rate in cancer patients receiving immunotherapy [20]. Stemness indices are used to evaluate the similarity of tumor cells to stem cells [21]. This characteristic is mainly measured by using the mRNA expression-based stemness index (mRNAsi) as well as the epigenetic regulation-based index (EREG-mRNAsi). The ranges of mRNAsi and EREG-mRNAsi are from 0 to 1, where higher values indicate a high resemblance of tumor cells to stem cells [22]. Primary undifferentiated tumors have a greater ability to migrate aggressively or form distant metastases, ultimately resulting in tumor progression [23]. It is basic to disclose the alteration of the TME and immunization checkpoints, and identify predictive biomarkers for immunotherapy response and stemness indices for promoting the investigation to develop novel therapeutic strategies in GBM.

Based on genetic transcription features, GBM can be distinguished as five subtypes(classical, CL; mesenchymal, MES; neural; proneural, PN; proliferative) [24]. Different subtypes have significantly different prognosis. The results of a related study showed that patients with the proneural subtype have a better prognosis, whereas patients with the mesenchymal subtype show a significantly more aggressive and poor prognosis [25–27]. In addition, different subtypes of glioblastoma have different epigenomic markers, and some of them have shown prognostic and predictive values. Thus, further typing of GBM will help to understand the differences between subtypes of GBM, which is important for developing more personalized and accurate treatment plans to combat GBM.

In the study, we analyzed the differential expression of cell senescence-associated genes in GBM. Then, we constructed a risk scoring model of cell senescence-associated genes. According to the risk score model, the clinical prognostic factors, immune checkpoints, mutations of cell senescence-associated genes, and drug sensitivity in patients with GBM were discussed. Finally, Through GBM data in the CGGA database, TCGA database and pan-cancer analysis further verified the rationality and reliability of the risk scoring model.

## Materials and methods

### Data Collection

The RNA-seq transcriptome data and clinical information (including gender, age, IDH status, CIMP status, etc.) of GBM were downloaded from the cancer genome atlas (TCGA, https://portal.gdc.cancer.gov/), the Chinese Glioma Genome Atlas (CGGA, Home | CGGA - Chinese Glioma Genome Atlas) and the GlioVis [GlioVis - Visualization Tools for Glioma Datasets (cnio.es)]. Somatic mutation counts and copy number variation (CNV) also were downloaded from the TCGA database. 279 cell senescence-associated genes were acquired from the cell senescence gene database (CellAge, https://genomics.senescence.info/cells/). Significant differential expression of cell senescence-associated genes was set at FDR < 0.05 and |log2FC|≥2. 39 differentially expressed genes that were related to cell senescence were finally obtained in this study.

### Characteristics of the cell senescence-associated genes

Firstly, We analyzed the correlated regulatory relationships between 39 cell senescence-associated genes by using the R package “igraph”. In the meantime, the somatic mutation prevalence, the copy number variation, and the genetic locus of cell senescence-associated genes were analyzed. We further dissected the differences in the expression of cell senescence-associated genes between tumor cells and normal cells by applying variance analysis. We also use the same research method to analyze the profile of 39 cell senescence-associated genes in different expression types of GBM (including mesenchymal,; classical; and proneural). Combining GBM patient survival, 39 cell senescence-associated genes were analyzed using univariate Cox regression and forest plots were drawn.

### Immunohistochemistry (IHC) staining

We chose the most representative genes with a positive or negative correlation with the prognosis of GBM patients as the research objects, whose IHC staining was downloaded from the Human Protein Atlas (HPA: http://www.proteinatlas.org/) and analyzed. This allowed us to assess differences in cell senescence-associated genes expression at the protein level.

We collected section from paraffin-embedded tissues of human glioma and peritumor. We dewaxed and dissociated the sections and rehydrated sections. After heating in tris-EDTA buffer, we blocked slides using 5% gout serum and incubated slides with primary antibody (PTTG1, 1:500, #ab128040; Abcam) (MYC, 1:100, #ab32072; Abcam) at 4 °C overnight. Then the slides were incubated with secondary antibody and the images were captured using a Leica DM 2500 microscope.

### Cell culture

GBM cell line U251 was purchased from the Chinese Academy of Sciences (Shanghai) culture bank. Cells were cultured in Dulbecco’s modified Eagle’s medium (DMEM; Thermo Fisher Scientific; Waltham, MA, USA) supplemented with 10% fetal bovine serum (FBS; Thermo Fisher Scientific, USA). Cells were maintained at 37 °C in a humidified chamber containing 5% CO_2_.

### PTTG1 and MYC silencing

siRNAs targeting PTTG1 and MYC were synthesized (GenePharma; Shanghai, China). Lipofectamine™ 3000 reagent (Thermo Fisher Scientific; USA) was used to transfect siRNAs according to manufacturer’s protocol. Knockdown efficiency was evaluated by Western blotting. siRNA sequences are the following: si-PTTG1: 5′-GGGAGATCTCAAGTTTCAA-3′; and si-MYC: 5′-GCUUGUACCUGCAGGAUCUTT-3′.

### Western blotting

Harvested cells are thermally denatured in the RIPA cell lysis buffer. The protein lysate was run on SDS-PAGE and the protein was transferred to the PVDF membrane. Blots are cultured with primary antibodies against PTTG1 (#ab128040; Abcam), MYC(#ab32072; Abcam); GAPDH (Cell Signaling Technology; Danvers, MA, USA). Specific proteins were detected with enhanced chemiluminescence (ECL, Millipore, Bedford, MA, USA). GAPDH was used as the loading control.

### 5-ethynyl-2′-deoxyuridine (EdU) cell proliferation assay

An EdU cell proliferation assay kit (RiboBio, #C10310-1; Guangzhou, China) was used to measure the glioma cell proliferation according to the manufacturer’s protocol. The glioma cells were incubated in 250 µl EdU solution for 2 h. Cells were fixed in 4% paraformaldehyde for 15 min, 0.4% Triton X-100 penetrability for 10 min, and incubated with 250 µl Apollo^®^ reagent for 30 min. Then the cells were stained with Hoechst 33,342 for 30 min. The ratio of EDU positive cells (red) to the total number of Hoechst 33,342 positive cells (blue) was used as cell proliferation rate.

### 3D tumor spheroid invasion assay

Glioma cells were seeded into a 3D culture qualified 96-well spheroid-formation plate (5 ×10^3^ cells/well) and incubated in the spheroid-formation matrix (Trevigen, Gaithersburg, MD, USA) and DMEM containing 10% FBS for 72 h. When the spheroids grew to a diameter of > 200 mm, the invasion matrix (Trevigen, Gaithersburg, MD, USA) was infused. The spheroids at 24 h were regarded as a reference point for measuring the area invaded by the sprouting cells. Spheroids were imaged every 24 h using a Leica microscope.

### Unsupervised clustering for cell senescence-associated genes in GBM

We explored potential molecular subtypes of GBM patients obtained from GlioVis datasets, which were distinguished into the gene.clusters.C1(C1), gene.clusters.C2(C2) and gene.clusters.C3(C3), by using the Consensus Cluster Plus (CC) R package based on unsupervised cluster analysis. The survival differences of different potential molecular subtypes of GBM patients were analyzed through the log-rank test to test the rationality of distinguishing different potential subtypes.

### Characteristic differences of C1, C2, and C3 subtypes

To understand the variability in the degree of similarity between different subgroups of GBM tumor cells and stem cells, we examined the stemness index of GBM in the C1, C2 and C3 subtypes separately and draw the box diagram. The mutual relationships of three potential subtypes, clinical typology and presence or absence of cytosine-phosphate-guanine (CpG) island methylator phenotype (G-CIMP) were demonstrated by the Sankey diagram. Single-sample gene set enrichment analysis, ESTIMATE as well as CIBERSORT were leveraged to quantify the TME in three subtypes. Additionally, three different possible molecular subtypes of GBM patients’ immunological checkpoints, immune cell infiltrate and immune inhibitors and/or stimulators were examined. Gene ontology biological processes (GOBP) and Kyoto Encyclopedia of Genes and Genomes (KEGG: http://www.genome.jp/kegg/pathway.html) pathways were used to annotate the three subtypes.

### Construction of the risk score

Weighted Gene Co-expression Network Analysis (WGCNA) was leveraged to recognize differentially expressed genes relevanted in the prognosis of GBM. Then, the univariate Cox regression analysis, least absolute shrinkage and selection operator (LASSO) regression and multivariate Cox regression analysis were exerted to gain the cell senescence-associated genes for predicting survival and prognosis of GBM. The risk score formula is shown below:

Risk Score=$${\sum }_{\text{j}=1}^{\text{n}}\text{C}\text{o}\text{e}\, \text{g}\text{e}\text{n}\text{e}\text{j}\times \text{E}\text{x}\text{p} \,\text{o}\text{f} \,\text{g}\text{e}\text{n}\text{e}\text{j}$$  

The Coe (genej) was the abbreviation of the coefficient of genes and Exp (genej) was the expression of genes.

The risk score was assigned systematically randomly into the train set and test set in a 1:1 ratio and went for intra-group validation. Then, the reliability of the risk score was assessed through the Log-rank test of the train set and test set. The receiver operating characteristic curve (ROC curve) is a comprehensive index reflecting the continuous variables of sensitivity and specificity, which is mainly through the area under the ROC curve (AUC) [28]. The value of AUC is between 0.5 and 1.0. When AUC > 0.5, the closer the AUC is to 1, the better the diagnostic effect. When AUC = 0.5, the diagnostic method is completely ineffective[29]. In this study, we assessed the specificity and sensitivity of the risk score model in predicting the survival and prognosis of GBM patients through the value of AUC. We then analyzed the survival situation of GBM patients in high- and low- risk scores and the differences in cell senescence-associated genes expression in different risk scores were assessed. Further, we obtained the clinical prognostic factors associated with the prognosis of GBM patients by combining risk scores and clinical characteristics.

### Nomogram construction and immune checkpoint acquisition

The potential interrelationships regarding the three potential subtypes, clinical typing, the G-CIMP or NON-G-CIMP and risk scores also were revealed by the Sankey diagram. The nomogram, combining prognostic characteristics and clinical characteristics, was performed by leveraging the R package “RMS”. We evaluated the risk score models well as the nomogram by calibration curves and time-dependent ROC curves. Also, we explored the statistical significance of risk scores with different clinical prognostic factors. The single-sample gene set enrichment analysis, ESTIMATE as well as CIBERSORT were shafted to quantify the difference in TME in high- and low-risk scores. Due to the application prospect of immunotherapy, the differences in immune checkpoints, immune cell infiltration and immune inhibitors (or stimulators) in different risk scores of GBM patients were also measured.

### Verification of the risk score

The risk scoring model was validated by the GBM dataset obtained from the CGGA database and TCGA database and the clinical prognostic factors were analyzed. Then, we assessed the specificity and sensitivity of the risk score model in predicting the survival and prognosis of patients with glioblastoma through the value of AUC again. Next, we constructed nomograms and verified them with calibration curves by leveraging the R package “RMS”. At the same time, the tumor mutational burden (TMB) of high- and low-risk scores was analyzed based on The TCGA database by leveraging the R package “ maftools”.

### Pan-cancer analysis and the drug sensitivity

The discrepancy between TMB, microsatellite instability (MSI), and CD274 in 32 different cancer types was analyzed and defined their differences and similarities based on the risk score. Additionally, We explored the relatedness between risk scores and TME as well as stemness indices in pan-cancer. The expression information of different cell lines was obtained from Broad Institute Cancer Cell Line Encyclopedia (CCLE, https://portals.broadinstitute.org/ccle/about).

Then, the percent weight of binary response and immune phenotype in high- and low-risk scores were measured. Tumor neoantigens are not only highly specific but also strongly immunogenic, which are usually expressed only in tumor tissues [30, 31]. The prognosis of GBM patients combining risk scores and tumor neoantigens was predicted by survival analysis and plotting the survival curve. The drug response information and drug targeting pathways were analyzed by spearman correlation analysis to acquire drugs associated with risk scores, which were collected from Genomics of Drug Sensitivity in Cancer (GDSC, https://www.sanger.ac.uk/tool/gdsc-genomics-drug-sensitivity-cancer/).

### Statistical analysis

**Fig. 1:**
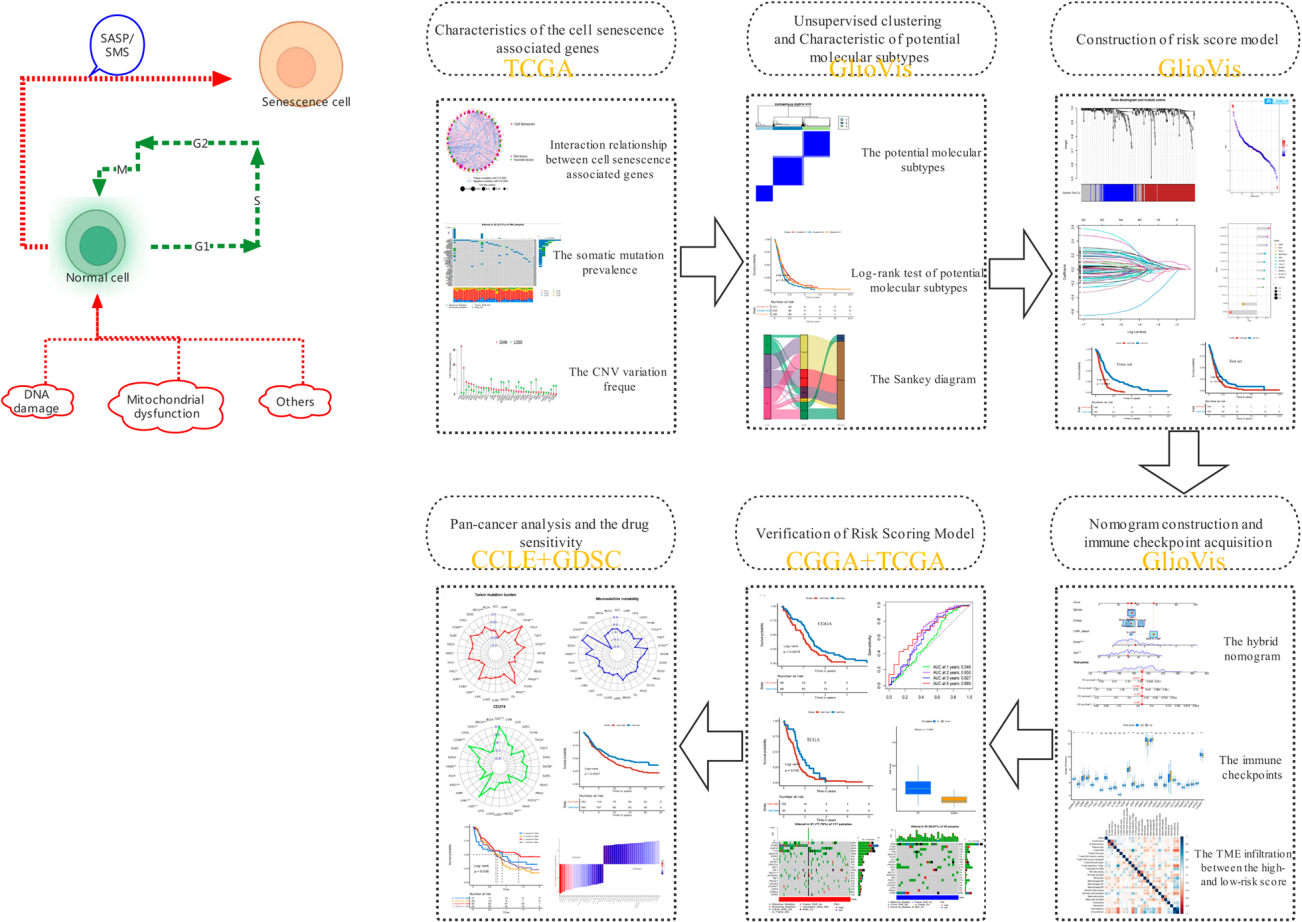
The flowchart of this study

In the present experiment, the statistical analyses were performed in R software (version 4.1.3). Related R packages including “ pheatmap,” “ edgeR,” “ glmnet,” and “ forestplot” and other related R packages were obtained from Bioconductor packages or R packages and some of the study results were visualized and presented by the Emerging biomedical data visualization toolkit ( Hiplot: https://hiplot-academic.com). For each analysis, statistical significance was set at *P* < 0.05. In this study, the flowchart was presented in Fig. 1.

## Results

### The characteristics of cell senescence-associated genes

**Fig. 2:**
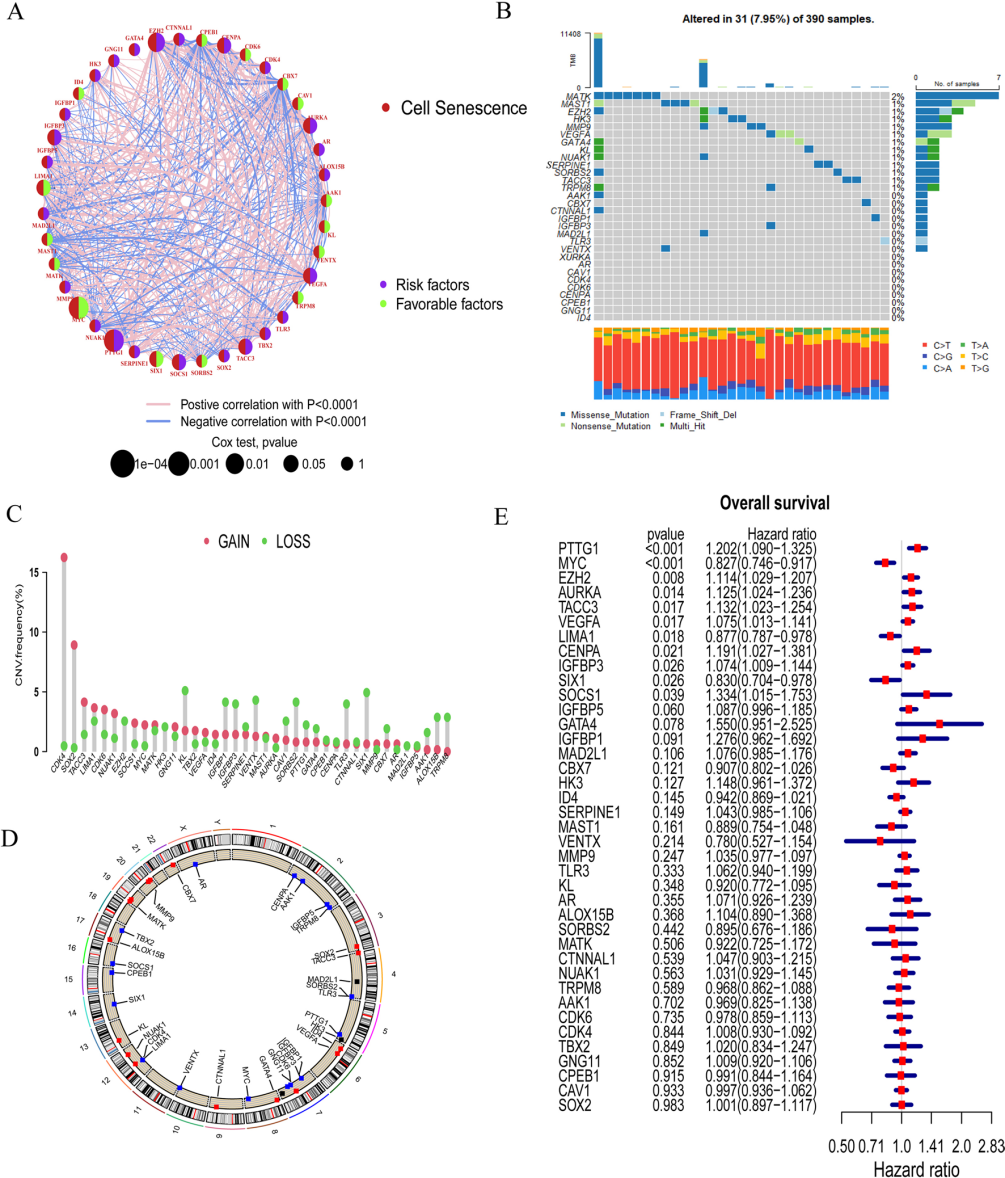
The characteristics and expression of differences of cell senescence-associated genes in GBM **A** Network diagram showing the interaction of 39 cell senescence-associated genes in GBM. The size of the circles indicates the p-value of each gene on survival prognosis. Purple represents risk factors and green dots represent favorable factors. The thickness of the lines indicates the correlation values between genes. The red and blue lines represent positive and negative correlations of gene regulation, respectively. **B** Mutation prevalence of 39 cell senescence-associated genes in GBM. **C** The copy number variation (CNV) of 39 cell senescence-associated genes in GBM. **D** The localization of 39 cell senescence-associated genes the on TCGA-GBM 23 chromosomes. **E** The effect of 39 cell senescence-associated genes on the overall survival for GBM patients

A total of 39 cell senescence-associated genes were included in this study through difference analysis (Additional file 9: Table. S1). We analyzed the regulatory relationships associated with the expression of 39 cell senescence-associated genes in GBM patients, for example, EZH2 positively regulates PTTG1 expression, while PTTG1 has a significant positive effect on TACC3 expression, and the result is presented in Fig. 2A. In the meantime, to deepen our understanding, we researched the somatic mutation prevalence of cell senescence-associated genes among GBM. Among them, a total of 13 out of 39 cell senescence-associated genes have somatic mutations and the MATK had the highest mutation rate, which is 2%, and mainly missense mutations (Fig. 2B). The analysis straightforwardly represented the differences in mutation profiles of different cell senescence-associated genes in GBM. Moreover, the investigation of 39 cell senescence-associated genes exhibited that CNV-related mutations were widespread. CKD4, SOX2, TACC3, CDK6, NUAK1, SOCS1 and MYC showed widespread CNV amplification, while EZH2, HK3, KL, IGFBP1, IGFBP3, VENTX, CAV1, SORBS2, PTTG1, GATA4, TLR3, SIX1, CBX7, AAK1, ALOX15B and TRPM8 had CNV deletions (Fig. 2C). Meanwhile, the localization of the 39 cell senescence-associated genes on TCGA-GBM 23 chromosomes was determined (Fig. 2D). The expression of most cell senescence-associated genes in GBM tissues was different from that in normal tissues. Additionally, we found that the expression of 31 cell senescence-associated genes significantly varied among three subtypes (CL, MES and PN) of GBM (Additional file 1: Fig. S1 and Additional file 2: Fig. S2). In an exploration of the effect of 39 cell senescence-associated genes on the overall survival (OS) of GBM patients, we found that downregulation of MYC, LIMA1 and SIX1 expression and upregulation of PTTG1, EZH2, AURKA, TACC3, VEGFA, CENPA, IGFBP3 and SOCS1 expression exhibited a statistically significant effect on the overall survival of GBM patients (Fig. 2E).

**Fig. 3:**
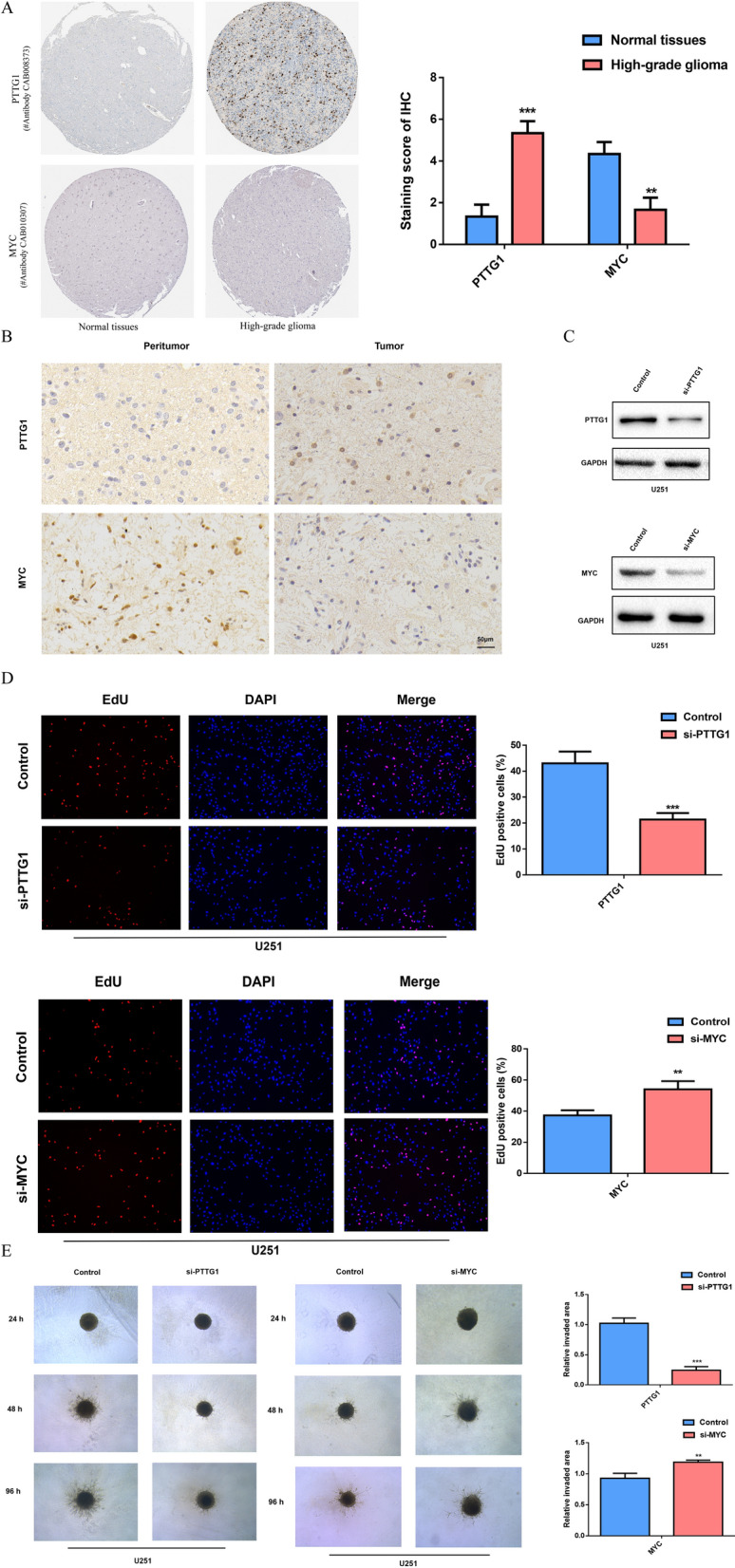
Validation of PTTG1 and MYC expression and their function in glioma.** A** The expression level of PTTG1 and MYC in normal tissue and glioma from the HPA. **B** Representative images of IHC staining for PTTG1 and MYC in tumor and peritumor tissue (scale bar: 50 mm). (**C**) Western blot was used to verify the efficiency of PTTG1 and MYC downregulation. **D** The result of EdU assay 48 h after transfection was showed. **E** Representative images showing invading spheroids in the 3D invasion assay for U251 cells transfected with si-RNA and control evaluated at 24 h, 48 and 96 h

Furthermore, the IHC staining of PTTG1 and MYC was downloaded from the HPA. The result showed that the protein encoded by PTTG1 was higher expressed in glioma than normal tissues (#Antibody CAB008373). The level of MYC had lower expression in glioma than normal tissues (#Antibody CAB010307) (Fig. 3A). IHC staining was performed to detect the representative PTTG1 and MYC protein levels in gliomas and peritumor tissues (15 cases) of The Second Hospital of Shandong University. Consistent with the previous results, the protein level of PTTG1 was significantly increased in glioma in comparison to peritumor tissue, while the level of MYC was decreased in tumor (Fig. 3B).

To explore the effect of PTTG1 and MYC on glioma cell survival and invasion, we transfected cells with siRNA to downregulate PTTG1 and MYC. Western blot was used to verify the efficiency (Fig. 3C). Then EdU assays were performed. The downregulation of PTTG1 resulted in significant decreases in the percentage of EdU positive cells in U251 cells 48 h after transfection, while knockdown of MYC showed the opposite trend (Fig. 3D). In order to investigate the influence of PTTG1 and MYC on the invasion of glioma cells, we conducted a 3D collagen spheroid invasion assay. Silencing PTTG1 reduced the area invaded by U251 spheroids relative to controls, while knockdown of MYC increased the area. (Fig. 3E). These results suggested that PTTG1 and MYC silencing significantly influenced glioma cell activity in vitro.

### Characterization and TME in different potential molecular subtypes

**Fig. 4:**
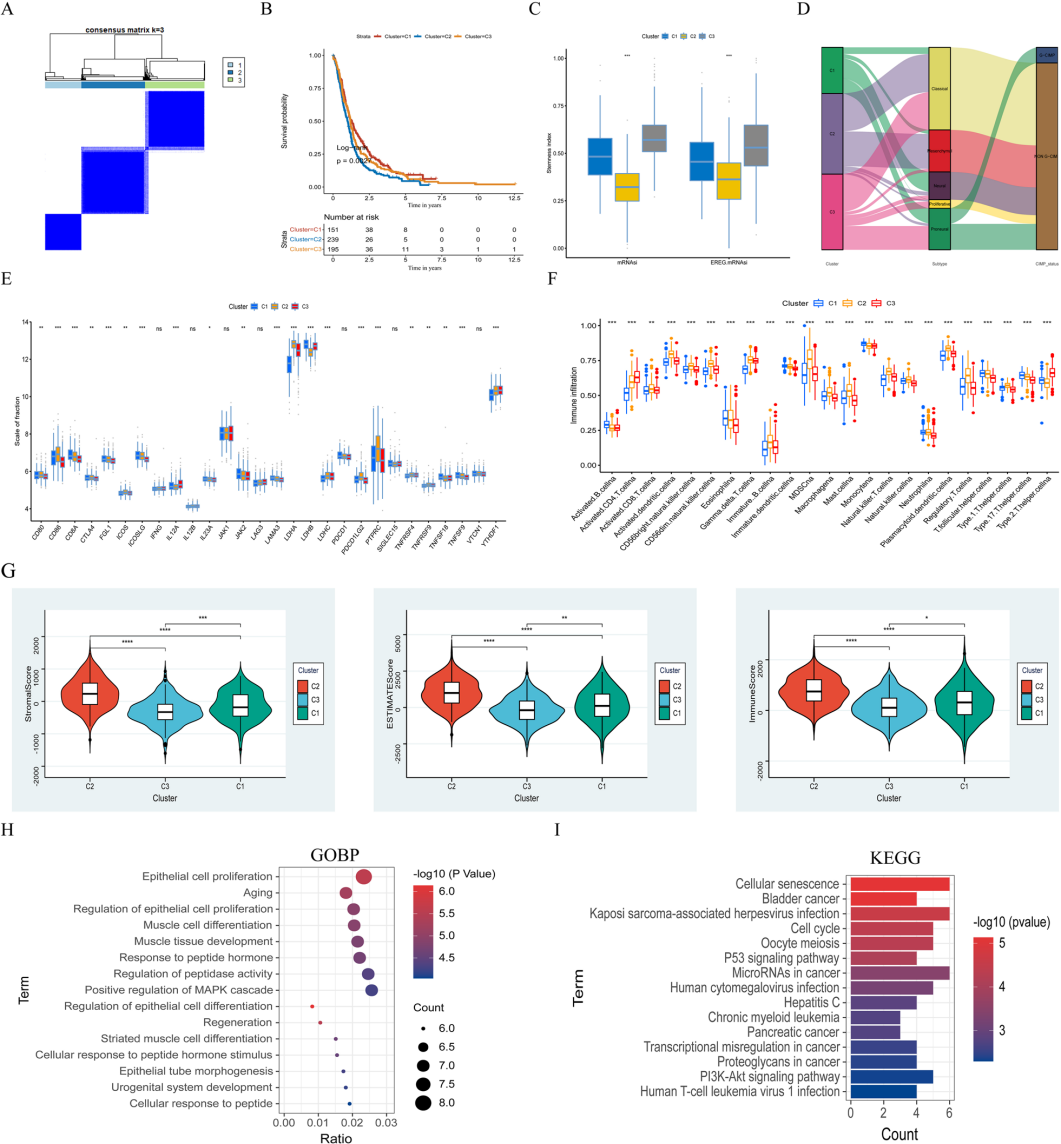
Characterization and TME in different potential molecular subtypes. **A** The GBM patients (n = 585) were divided into 3 potential molecular subtypes (C1, C2 and C3) by using the ConsensusClusterPlus (CC). **B** Log-rank test for the C1 (151 cases), C2 (239 cases) and C3 (195 cases) cohorts (*p* = 0.0027). **C** The stemness index difference between the 3 subtypes. (**D**) The Sankey diagram is about the relationship between 3 subtypes, clinical typing, and cytosine-phosphate-guanine (CpG) island methylator phenotype (G-CIMP). **E** The regulatory role of cell senescence-associated genes on immunization checkpoint expression in GBM tumors. **F** The abundance of each TME-infiltrating cell in C1, C2, and C3 clusters. **G** The differences in stromal score ESTIMATE score and immune score between the 3 potential subgroups. GOBP **H** analyses and KEGG analyses **I** for cell senescence-associated genes

The unsupervised cluster analysis was used to classify the GBM patients which were the GlioVis dataset. 3 potential subtypes, gene. cluster1 (C1), gene.cluster2 (C2) and gene.cluster3 (C3) were acquired (Fig. 4A). The result of the Log-rank test showed the difference in GBM patient survival between 3 potential molecular subtypes (*P* = 0.0027) (Fig. 4B), which indicated that the GBM patients in C1, C2 and C3 subtypes were different and that confirmed the 3 subtypes distinction is reasonable. The mRNAsi and EREG-mRNAsi of the C3 cluster were closer to the 1 in the stemness index analysis, which suggests that GBM in C3 has a notable resemblance to stem cells (Fig. 4C). However, further research is necessary, as some studies have shown that higher indices appear to be directly associated with the degree of progression and poor prognosis for a variety of cancers, which is not consistent with the results of this study. In the Sankey diagram, the interrelationship between 3 subtypes and clinical typology, and G-CIMP/NON-G-CIMP was identified (Fig. 4D). In the exploration of the TME between the 3 subtypes, we found discrepancies in immunization checkpoints and immune cell infiltration. For instance, the expression of CD80, CD86, LDHA and PTPRC were abundant in C2 subtypes (Fig. 4E). And T cell regulatory, T cell CD8, B cells naive, dendritic cell activated, and other immune cells were also remarkably abundant in C2 subtypes. While NK cells activated and macrophages M2 were a significant expression in C1 subtypes (Fig. 4F). Meanwhile, The violin diagram demonstrates the differences in stromal score ESTIMATE score and immune score between the 3 potential subgroups (Fig. 4G). In the GOBP and KEGG analysis of the biological pathways of GBM, it was found that the senescence pathway plays a major role in GBM and the results are shown in Fig. 4H , I.

### Construction of the risk score and Acquisition of clinical prognostic factors

**Fig. 5:**
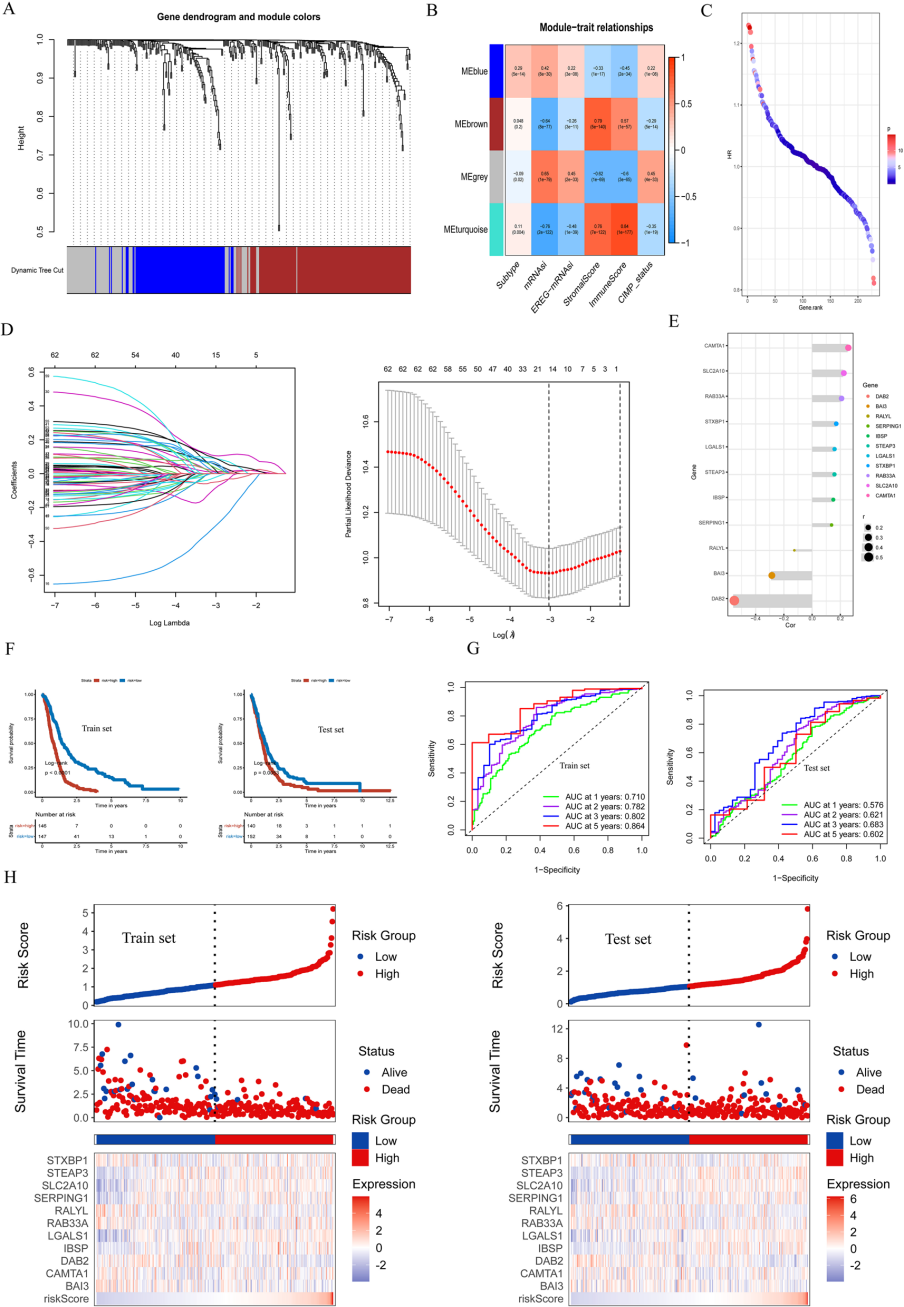
Constructing risk score models. **A** Weighted gene co-expression network analysis (WGCNA) based on gene expression data identified gene modules with highly synergistic changes. **B** The heatmap of module-trait relationships. (**C**) Univariate Cox regression analysis of 228 genes relevant to GBM prognosis. **D** The least absolute shrinkage and selection operator (LASSO) method of cell senescence-associated genes. **E** The multivariate Cox regression analysis ultimately found 11 gene sets associated with prognosis to construct risk score. (**F**) Kaplan–Meier curves of the train set (*P* < 0.001, log-rank test) and test set (*P* = 0.0053, log-rank test). **G** Time-dependent receiver operating characteristics (ROC) of trainset and test set. **H** Correlations between survival status and risk score and gene expression status and risk score

The WGCNA operations were performed on the combined GBM dataset to obtain the key modules most relevant to the clinical features (Fig. 5A). A total of 228 differential cell senescence-associated genes were obtained, while co-expression modules were identified (Additional file 10: Fig. S10). According to the heatmap of module-trait relationships, the ME blue and ME turquoise modules demonstrate the highest pertinence with clinical features (Fig. 5B). Univariate Cox regression algorithm was exerted to, preliminary acquisition of 62 genes (Additional file 11: Fig. S11) associated with GBM prognosis and the HR and P values of the 228 cell senescence-associated genes were calculated, and the result is shown in Fig. 5C. Next, the LASSO algorithm and multivariate Cox regression analysis were applied to determine the prognostic gene set of GBM and ultimately found 11 gene sets (Fig. 5D and E). Finally, 11 cell senescence-associated genes were used to construct the risk score for predicting the survival and prognosis of GBM patients. The cohort of GlioVis-GBM patients was systematically randomized to distinguish the train set (n = 293) and the test set(n = 292). The Kaplan-Meier analyses evidenced that the GBM patients with high-risk scores in the train set (P < 0.0001) as well as the test set (P = 0.0053) corresponded with less favorable survival (Fig. 5F). The 1-, 2-,3-,5- year AUC of the train set were 0.710, 0.782,0.802 and 0.864, and of the test set were 0.576, 0.621, 0.683 and 0.602, respectively (Fig. 5G). In exploring the relationship between survival status and risk score, the survival rate of GBM patients gradually decreased as the risk score increased in both the train set as well as the test set (Fig. 5H). The multivariate Cox regression analyses were utilized to assess clinical independent prognostic factors for GBM patients. The results show that age was an independent prognostic factor for GBM patients in the train set as well as the test set (Additional file 3: Fig. S3 and (Additional file 4:  Fig. S4).

### The Hybrid Nomogram and TME

**Fig. 6:**
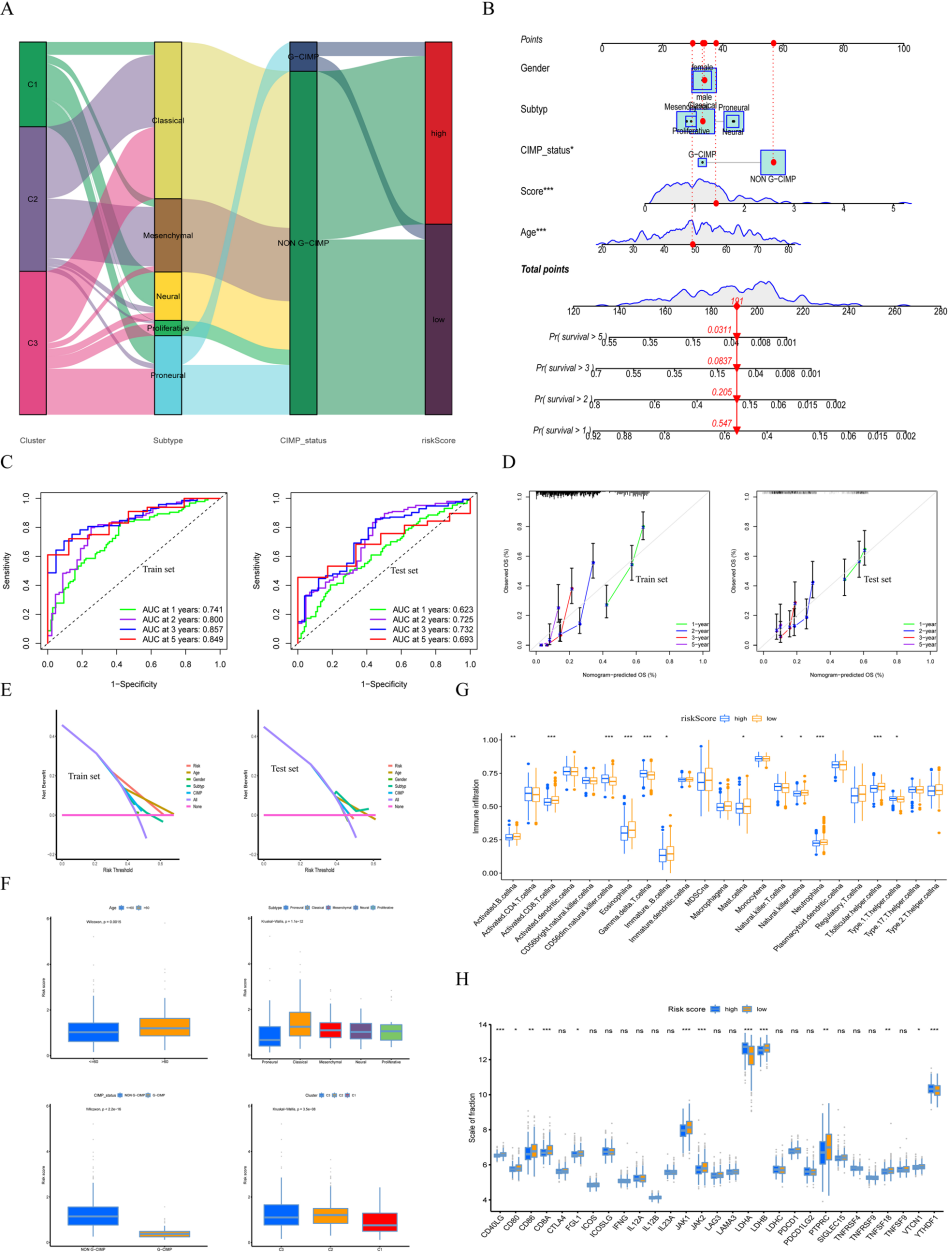
The hybrid nomogram and TME basing on risk scores in GBM. **A** The Sankey diagram is about the relationship between 3 subtypes, clinical typing, cytosine-phosphate-guanine (CpG) island methylator phenotype (G-CIMP), and risk score. **B** The hybrid nomogram integrated the clinical factors and risk scores. **C** The ROC of the train set and a test set containing a nomogram. **D** Validation of the ROC of the train set and a test set containing a nomogram. **E** The decision curve analysis (DCA) of trainset and test set containing nomogram. **F** The relationship between the risk score model and age, 3 subtypes, clinical typing and cytosine-phosphate-guanine (CpG) island methylator phenotype (G-CIMP). **G** The differences in the TME infiltration between the high- and low-risk score. **H** Expression of immune checkpoints among high and low GBM risk groups

The mutual relationships regarding the three potential subtypes, clinical typology, the G-CIMP or NON-G-CIMP and risk scores were reflected by the Sankey diagram (Fig. 6A). The hybrid nomogram can be used in the clinical administration of GBM patients due to its stable and accurate characteristics (Fig. 6B). The nomogram was also integrated into ROC to assessed the survival time and survival situation of the GBM patients. The 1-, 2-,3-,5- year AUC of the train set were 0.741, 0.800,0.857, and 0.849, and of the test set were 0.623, 0.725, 0.732 and 0.693 (Fig. 6C). The calibration curves were applied to evaluate and validate the ROC, and were shown in Fig. 6D. Meanwhile, the differentially expressed genes of the risk factors were displayed in Fig. 6E. In the valuation of the liaison between the risk score and different underlying subtypes of GBM patients, the Wilcoxon test revealed a remarkable discrepancy in the risk score between the C1, C2 and C3 and the statistical difference between the risk score and G-CIMP or NON-G-CIMP also was appearance. And also, the Wilcoxon test proved a variance of age in high-risk and low-risk scores (Fig. 6F). In the analysis of TME, several immune-related factors in the low-risk score were found to be notably abundant (Fig. 6G). About the immune checkpoint, CD40LG, CD8A, JKA1, JAK2, LDHB and others were more pronounced high expression in low-risk scores as well as LDHA and YTHDF1 were expressed in high-risk scores significantly (Fig. 6H).

### Validation of the risk score

**Fig. 7:**
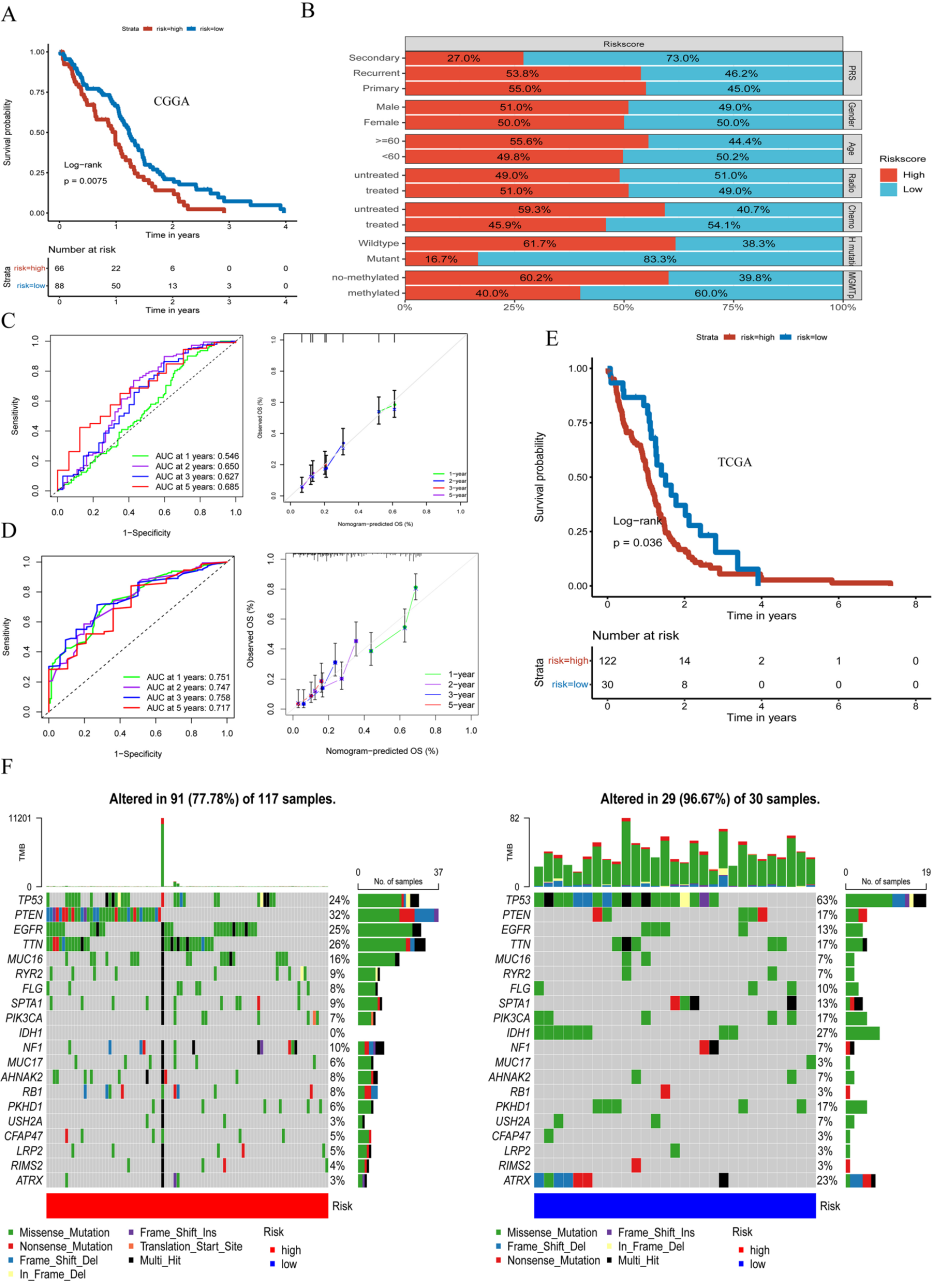
Validation of the risk score. (**A**) Kaplan–Meier curves of the risk score based on CGGA datasets (*P* = 0.0075, log-rank test). **B** The percent weight of different clinical prognostic factors of the high- and low-risk scores. **C** The AUC of the 1, 2, and 3-year survival rate of GBM and validation of the ROC of the risk score. **D** The AUC and validation of the ROC of the risk score containing nomogram. (**E**) Kaplan–Meier curves of the risk score based on TCGA datasets (*P* = 0.036, log-rank test). **F** Waterfall plot showing tumor mutational burden (TMB) presented by those with high-risk scores and low-risk scores

Apply the same arithmetic algorithm to construct the risk scoring model whose samples were obtained from CGGA datasets. Then, the Kaplan-Meier analyses ferreted out the expression of GBM patients with high-risk scores corresponded with poorer survival (*P* = 0.0075) (Fig. 7A). It is shown that the percentage weights of clinical prognostic factors in GBM patients with a high-risk score and low-risk score are in Fig. 7B. For example, in the high-risk score group, the percentage of GBM patients who were no-methylated was 60.2%. While in the low-risk score group, only 40% of GBM patients were no-methylated. After, we drew the hybrid nomogram according to the CGGA dataset (Additional file 5:  Fig. S5). The 1-, 2-,3-,5year AUCs which were combined risk score or nomogram score, were 0.564, 0.650,0.627, 0.685, and 0751, 0.747, 0.758, 0.717, respectively (Fig. 7C). We also passed the correction curve for verification (Fig. 7D). For further verification, we also constructed risk scoring models based on TCGA datasets and the same result was obtained by the Kaplan-Meier test (Fig. 7E). Furthermore, the map tools package was leveraged to the tumor somatic mutations presented in high- and low-risk scores respectively. The result shows that the low-risk score presented a wider range of TMB than the high-risk score (Fig. 7F). Meanwhile, the tumor somatic mutations of several genes are rarely observed in the low-risk group but frequently observed in the high-risk group, for example, PTEN. PTEN, which is the main negative regulator of the pI3k/Akt pathway and was a tumor suppressor gene with phosphatase activity closely related to tumorigenesis [32, 33]. But interestingly, TP3, the somatic mutation rate was as high as 63% in the low-risk group, which is a tumor suppressor gene with the function of regulating cell division and proliferation. The association of TP53 mutations with the development of a variety of tumors has been well documented [34, 35]. Therefore, further research is needed.

### Pan-cancer analysis and drug sensitivity

**Fig. 8:**
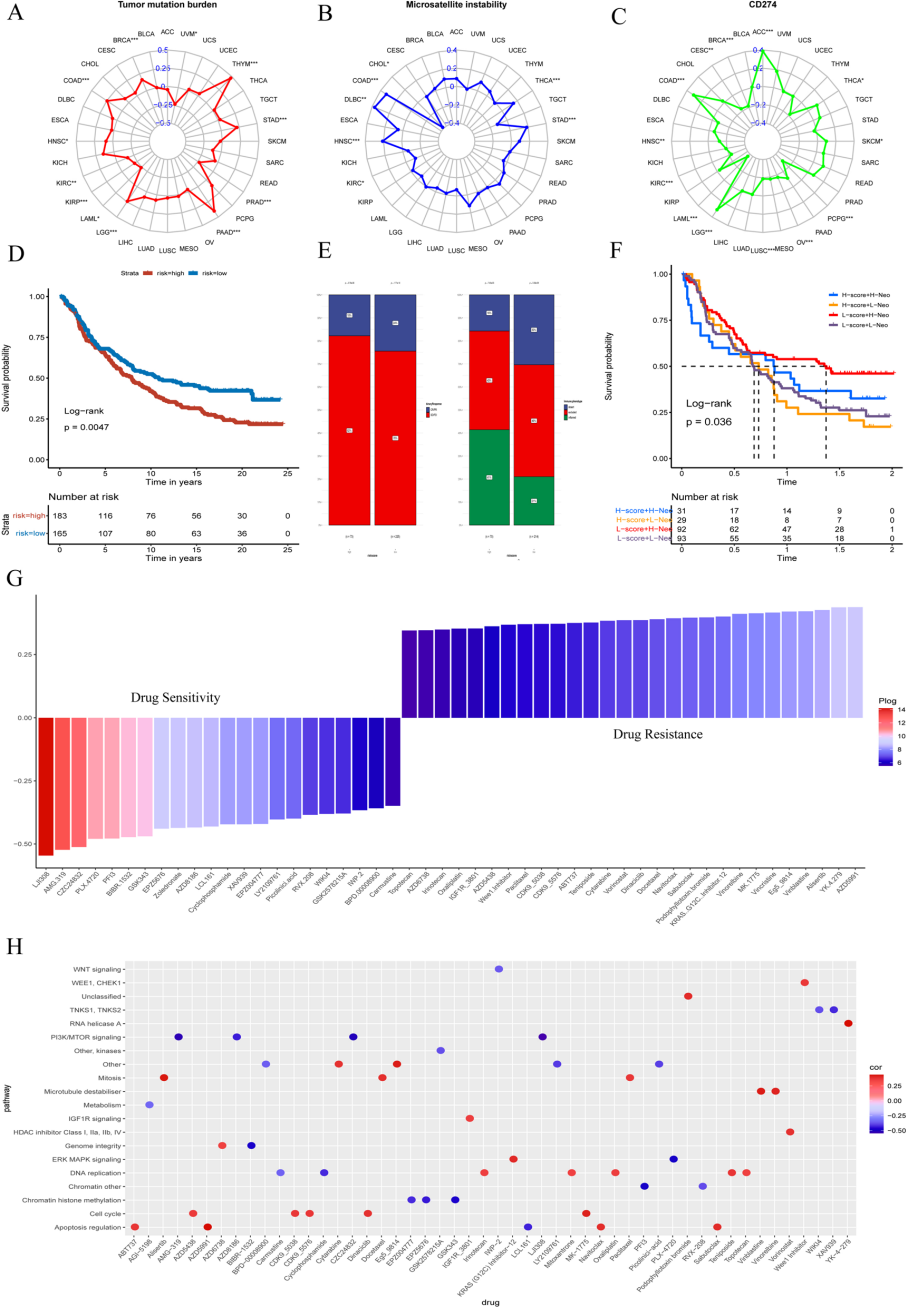
The pan-cancer analysis of 32 types of tumors and the drug sensitivity. Tumor mutation burden (TMB) **A**, microsatellite instability (MSI) **B**, and CD274 **C** of 32 types of tumors. **D** Survival analysis of the low- and high-risk score patient groups based on the IMvigor210 cohort. **E** The percentage of immunotherapy response and immunophenotype in high and low-risk scores. **F** The Kaplan-Meier test of GBM patients receiving ICB therapy by risk score combining tumor neoantigen burden. **E** Assessing drug sensitivity of GBM tumor based on the risk score. **F** Signal paths are targeted by drug sensitivity with the risk score. Blue (positive correlation) or red (negative correlation)

Pan-cancer analysis was used to assess similarities and differences in risk score models between different tumor types. We systematically evaluated TMB, MSI as well as the expression of CD274 among pan-cancer. The risk score was proactively correlated with TMB in BRCA, COAD, LGG, PAAD, STAD and THYM (P < 0.05), while the inverse correlation with TMB in KIRC, KIRP, LAML and UVM (P < 0.05) (Fig. 8A). For MSI, a positive correlation in STAD, DLBC, COAD, HNSC and THCA as well as a negative correlation in CHOL and KIRC, was defined (P < 0.05) (Fig. 8B). Additionally, the risk score was positively relevant to CD274 expression in ACC, COAD, HNSC, LGG, SKCM, THCA and negatively relevant with CD274 content in BRCA, CESC, HNSC, KIRC, LAML, LUSC, OV and PCPG(*P* < 0.05) (Fig. 8C). In addition, the mutual relationship between risk score and several immune cell infiltration as well as stemness indices were calculated, respectively (Additional file 6:  Fig S6 and  (Additional file 7: Fig. S7, (Additional file 8: Fig. S8). Using the immunotherapy cohort of advanced urothelial cancer (IMvigor210 cohort) to evaluate the impact of risk scores on predicting immunotherapy sensitivity. The Log-rank test also showed that GBM patients with a high-risk score were associated with poorer survival conditions (Fig. 8D). Then, integration risk scores were analyzed with immune checkpoint blockade (ICB) treatment studies. The results showed that the proportion of GBM patients of the high-risk score group in the response groups (CR and PR) was notably lower than in the low-risk score group, while the percentage of patients in the no/limited response groups (SD and PD) showed the contrary phenomena, pointing that the risk score could prove the response of GBM patients to ICB therapy. However, in exploring immune phenotypes in high- and low-risk scores, the desert phenomenon was more notable in the low-risk score, while the inflamed was seen more in the high-risk group (Fig. 8E). From combined risk score and tumor neoantigen burden correlation analysis, the GBM patients with low-risk scores together with a high neoantigen burden exhibited the most stretched-out survival time and the GBM patients with high-risk scores in connection with a low neoantigen burden had the worst survival situation (Fig. 8F). The Spearman correlation analysis was shafted to measure the value of the risk score to anticipate drug sensitivity for multiple types of cancer. Finally, 119 drugs for which the risk score and drug sensitivity significantly correlated, were obtained from the GDSC database. Subsequently, we selected the 50 most representative drugs for mapping. The risk score was the most significantly negatively sensitive to 5 drugs, including AZD5991, YK.4.279, Alisertib, Vinblastine and Eg5_9814; and the most significantly positively correlated with sensitivity to 5 drugs, including LJI308, AMG.319, CZC24832, PLX.4720 and PFI3 (Fig. 8G). Among them, the strongest drug sensitivity is LJI308. The study shows that LJI308 is a powerful selective inhibitor of RSK, which can inhibit the growth and proliferation of cancer stem cells [36]. In addition, the signal path targeted by the selected drugs was discovered. The relationship between drug sensitivity and risk score targeted the cell cycle, mitosis, microtubule, DNA replication and apoptosis regulation signaling were positive. On the contrary, drug sensitivity with negatively related to the risk score targeting the PI3K/mTOR signaling and chromatin histone methylation (Fig. 8H). In summary, the establishment of a risk score will be beneficial in exploring the facility and effective treatment strategies for GBM.

## Discussion

Cell senescence is a cellular defense mechanism that prevents the acquisition of unwanted damage in proliferating cells subjected to different stresses and is a permanent state of cell cycle arrest [37]. During embryogenesis and tissue remodeling, senescent process is required [38]. And senescent cells usually exhibit biological processes or biological pathways associated with senescent, including accumulation of lipofuscin, DNA damage foci, secretion of a large number of factors which were known as the senescence-associated secretory phenotype (SASP) and other changes [39–42]. It has been demonstrated that senescence can arrest tumor progression and can occur in different types of tumor cells [43, 44]. This tumor-suppressive function of senescence has provided emerging directions and paths for cancer therapy, a process termed pro-senescence therapy [45]. Therefore, this study focuses on the role of cellular senescence genes in drug resistance, immunotherapy, and the prediction of prognosis in GBM.

In our research, a total of 39 cell senescence-associated genes were eventually included. First, the characteristics of 39 cell senescence-associated genes represented by somatic mutations, and the CNV amplification were explored. Then, by exploring the impact of 39 cell senescence-associated genes on the overall survival of GBM patients, we identified 11 cell senescence-associated genes with statistically significant effects on the survival and prognosis of GBM patients (P < 0.005), which included PTTG1 and MYC. Currently, enormous shreds of evidence reveal that CS is an effective anti-tumor mechanism and proto oncogene-induced cell senescence is a barrier to preventing tumorigenesis as well as a target to treat tumors [46, 47]. PTTG1 in normal human fibroblasts can inhibit cell proliferation and result in several cell senescence-related events, including increasing SA-beta-galactosidase activity and SA-heterochromatin foci formation, decreasing BrdU incorporation [48]. Combined with the survival status of GBM patients, the results of univariate Cox regression analysis showed that PTTG1 and MYC have significant statistical differences. PTTG1-induced senescence was p53-dependent and telomerase-independent [49]. However, the high expression of PTTG1 in tumor cells can inhibit senescence, which has also been confirmed by many studies [50, 51]. For example, a study by Tong et al. showed that knocking down PTTG1 strengthened drug-induced senescence in colon carcinoma cells, confirming that PTTG1 functions to suppress drug-induced senescence [52]. Our study also showed the higher expression of PTTG1 in glioma and inhibition of PTTG1 decreased the proliferation and invasion of glioma cells. The MYC gene family includes c-Myc, N-Myc, and L-Myc [53]. Knocking down c-Myc signaling in human diploid fibroblasts (HDFs) was found to trigger telomere-independent senescence in human diploid fibroblasts (HDFs), which was mediated by cyclin-dependent kinase inhibitor p16 [54]. The amplification of c-Myc, N-Myc, and L-Myc was reported to be closely related to tumorigenesis and prognosis [55–59]. Whilst, c-Myc plays an important role in senescence and apoptosis [60, 61]. For example, in pancreatic and breast cancer cells, MYC expression products bind to and activate the promoters of the pro-apoptotic proteins BIM and BID, thereby facilitating the initiation of mitochondrial responses to apoptotic stimuli [62–64]. At the same time, there is also evidence that c-MYC levels progressively increase with age, leading to an age-dependent decrease in Nrf2 (nuclear factor erythroid 2 (NFE2)-related factor 2) signaling and adaptive homeostasis, thereby minimizing age-dependent cancer incidence [65]. Interestingly, in this study, high expression of MYC was beneficial for the prolonged survival of GBM patients. Therefore, further studies about the biological functions and biological pathways involved in MYC in GBM are necessary. Our studies indicated cell senescence-associated genes such as PTTG1 and MYC influenced the activities of glioma cell obviously.

Three potential molecular subtypes were obtained by classifying GBM patients using the unsupervised consistency clustering algorithm. After analysis and evaluation, there was a statistically obviously discrepancy in the survival status of GBM patients between the three subtypes. Meanwhile, differences in immune checkpoint and immune cell infiltration between subgroups were observed. Next, a risk score model based on differentially expressed genes was constructed to predict the survival and prognosis of GBM patients. At the same time, the potential possibility of immunotherapy in GBM was evaluated relying on the risk scoring model.

Current shreds of evidence have proved that immunotherapy may be a potential strategy for patients with GBM. However, the shortage of comprehension of the TME and immune cell infiltration in GBM results in undesirable therapeutic effects for patients receiving immunotherapy, which reveals the restrictions and deficiency of current clinical models of GBM. In this study, the risk score model for GBM prognosis based on the difference cell senescence-associated genes in expression was constructed to discover the value of the risk score in predicting the response of GBM to immunotherapy, and analyzing the differences in expression of immune-related cells in high and low-risk scores.

Meanwhile, we observed that patients who had lower risk scores exhibited prolonged overall survival in the ICB therapy and the utilization of this risk score model to predict the effect of immunotherapy in GBM patients was confirmed. In addition, an interaction between drug sensitivity and the risk score was observed by investigating the risk scores in GBM patients. The regulation of apoptosis and metabolism played an active role in the treatment of GBM. In contrast, the drug positively correlating with risk scores targets the cell cycle, mitosis, microtubule, DNA replication and apoptosis regulation signaling were positive. These findings suggest that inhibiting cell proliferation by promoting the expression of cell senescence-associated genes will contribute to the treatment of GBM.

## Conclusion

In this research, 39 cell senescence-associated genes were applied to systematically generate and assess risk scores for GBM and integrated these patterns with TME. The risk score models to predict prognosis as well as response to immunotherapy in GBM patients was established. The Systematic assessment of risk scores will extend our perception of GBM and will help to develop more individualized and refined treatment strategies. Moreover, this research will contribute to our understanding of the role of cell senescence-associated genes in GBM.

## Supplementary Information

**Additional file 1: Figure S1.**

**Additional file 2: Figure S2.**

**Additional file 3: Figure S3.**

**Additional file 4: Figure S4.**

**Additional file 5: Figure S5.**

**Additional file 6: Figure S6.**

**Additional file 7: Figure S7.**

**Additional file 8: Figure S8.**

**Additional file 9: Figure S9.**

**Additional file 10: Figure S10.**

**Additional file 11: Figure S11.**

## Data Availability

Not applicable.

## Abbreviations

GBM: Glioblastoma
TME: Tumor immune microenvironment
WGCNA: Weighted gene co-expression network analysis
LASSO: Least absolute shrinkage and selection operator regression
CNV: Copy number variation
TMB: Tumor mutation burden
SAMD: Senescence-associated mitochondrial dysfunction
MES: Mesenchymal
CL: Classical
P: Proneural
TCGA: The cancer genome atlas
CGGA: The Chinese glioma genome atlas
CC: The consensus cluster plus
ssGSEA: Single-sample gene set enrichment analysis
GOBP: Gene ontology biological processes
ROC: Receiver operating characteristic
MSI: Microsatellite instability
CCLE: Broad institute cancer cell line Encyclopedia
GDSC: Drug sensitivity in cancer
CpG: Cytosine-phosphate-guanine
G-CIMP: Cytosine-phosphate-guanine island methylator phenotype
ICB: Immune checkpoint blockade
DCR2: Decoy death receptor 2
SASP: Senescence-associated secretory phenotype
