# Supplementary figures and images for: Cell senescence-associated genes predict the malignant characteristics of glioblastoma
Source: Cancer Cell Int. 2022 Dec 16;22:411. doi: 10.1186/s12935-022-02834-1 (PMC9758946; doi:10.1186/s12935-022-02834-1)

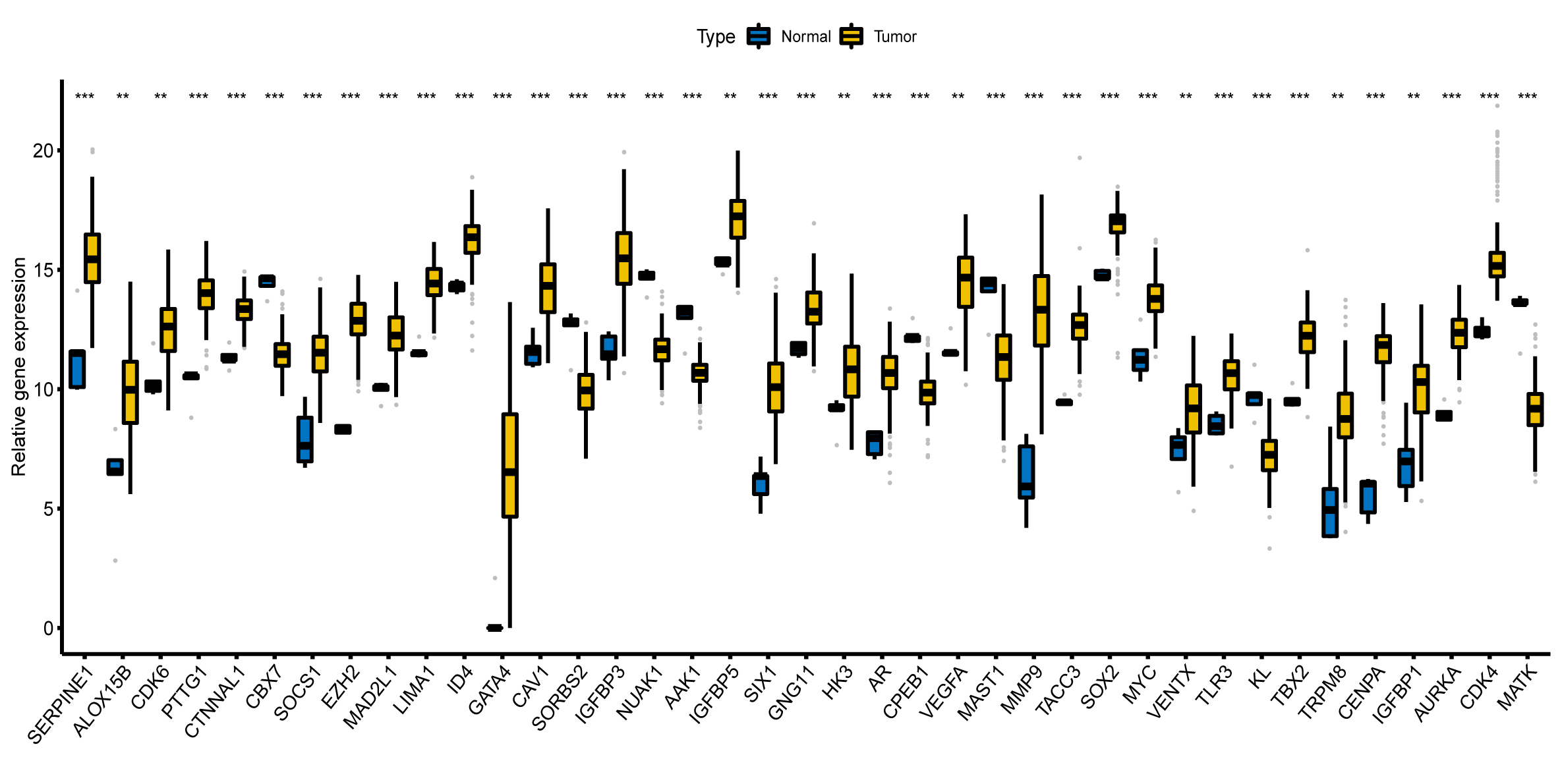

Supplement: Supplementary file 1 — Additional file 1: Figure S1. [file 12935_2022_2834_MOESM1_ESM.jpg]

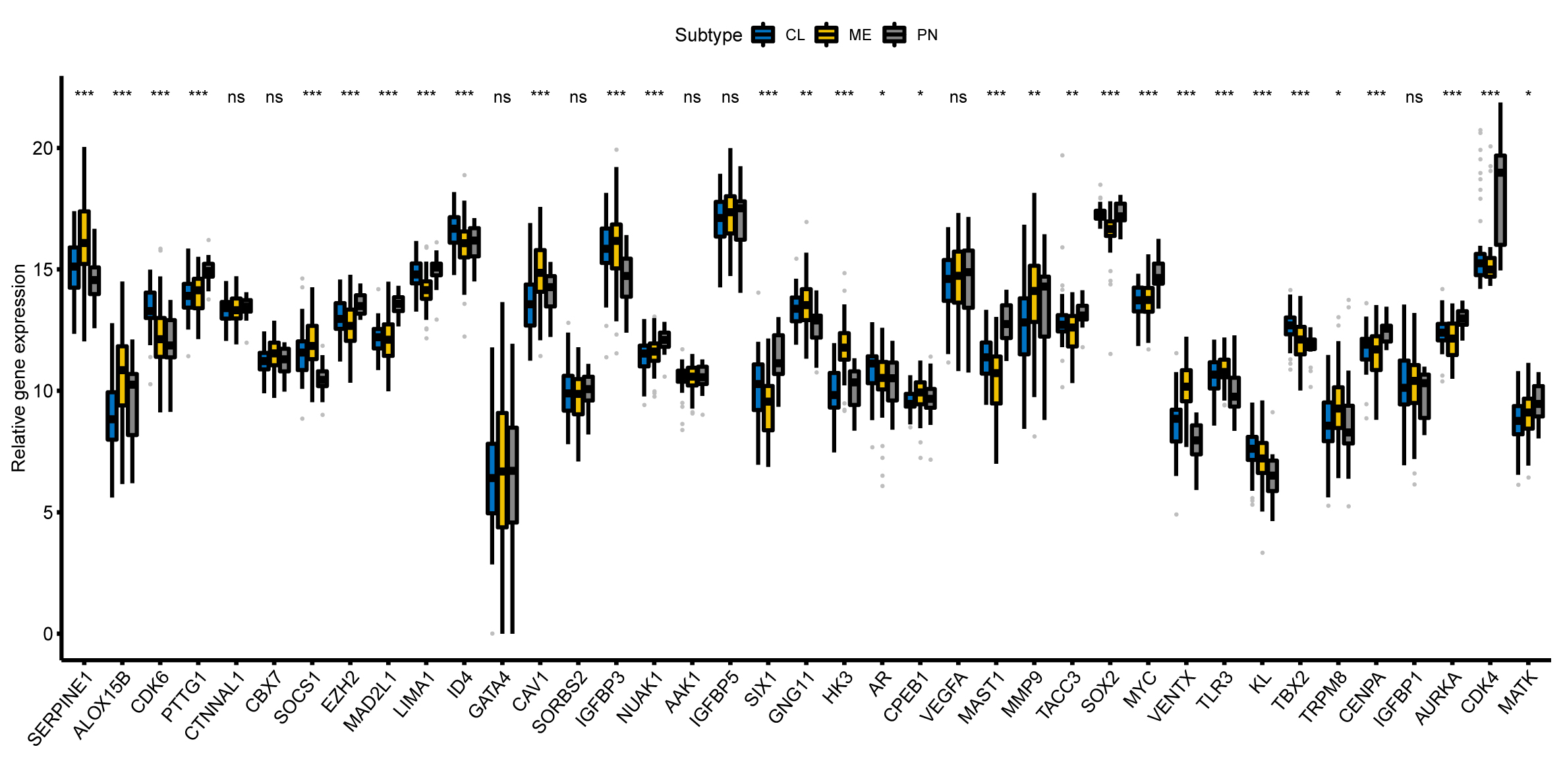

Supplement: Supplementary file 2 — Additional file 2: Figure S2. [file 12935_2022_2834_MOESM2_ESM.jpg]

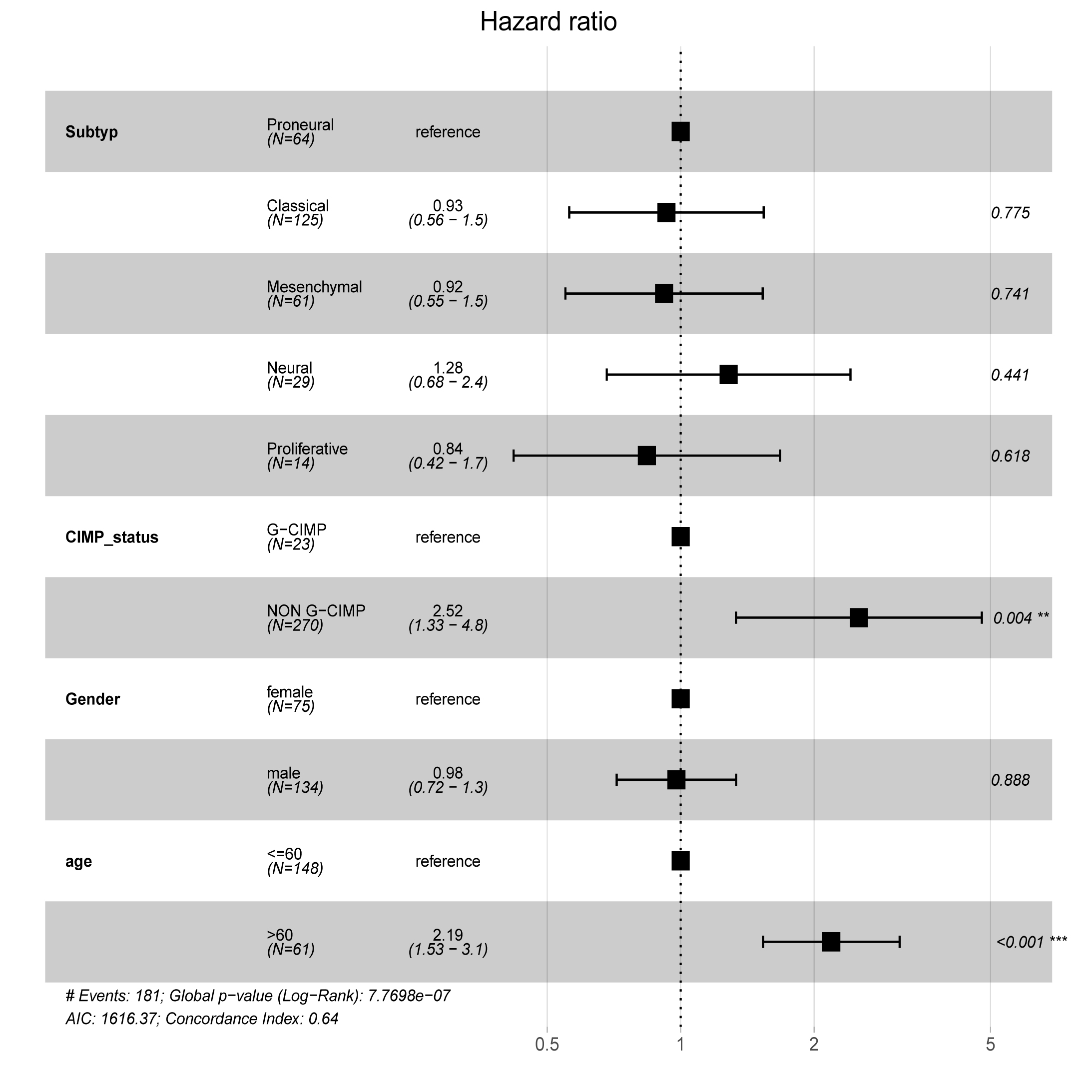

Supplement: Supplementary file 3 — Additional file 3: Figure S3. [file 12935_2022_2834_MOESM3_ESM.jpg]

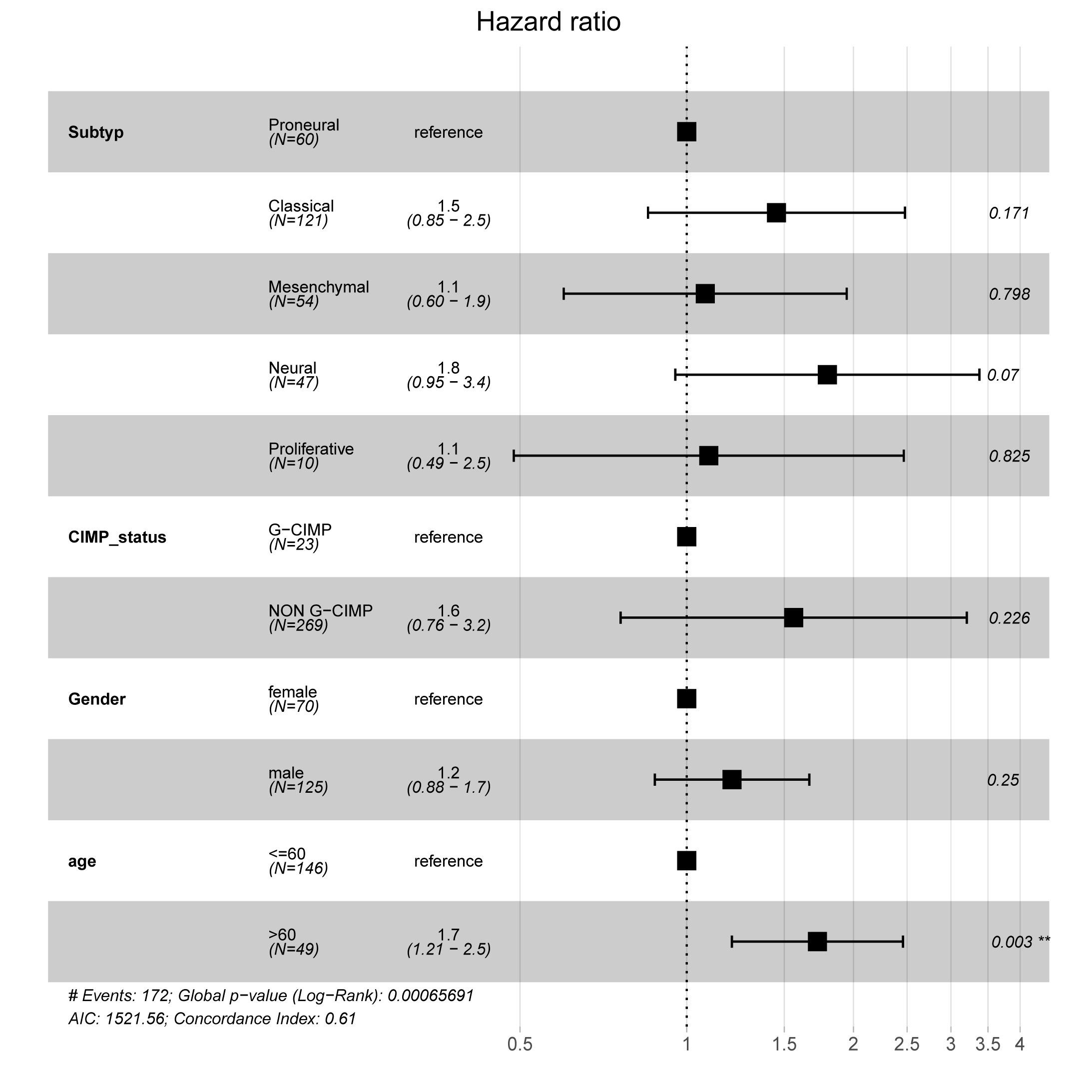

Supplement: Supplementary file 4 — Additional file 4: Figure S4. [file 12935_2022_2834_MOESM4_ESM.jpg]

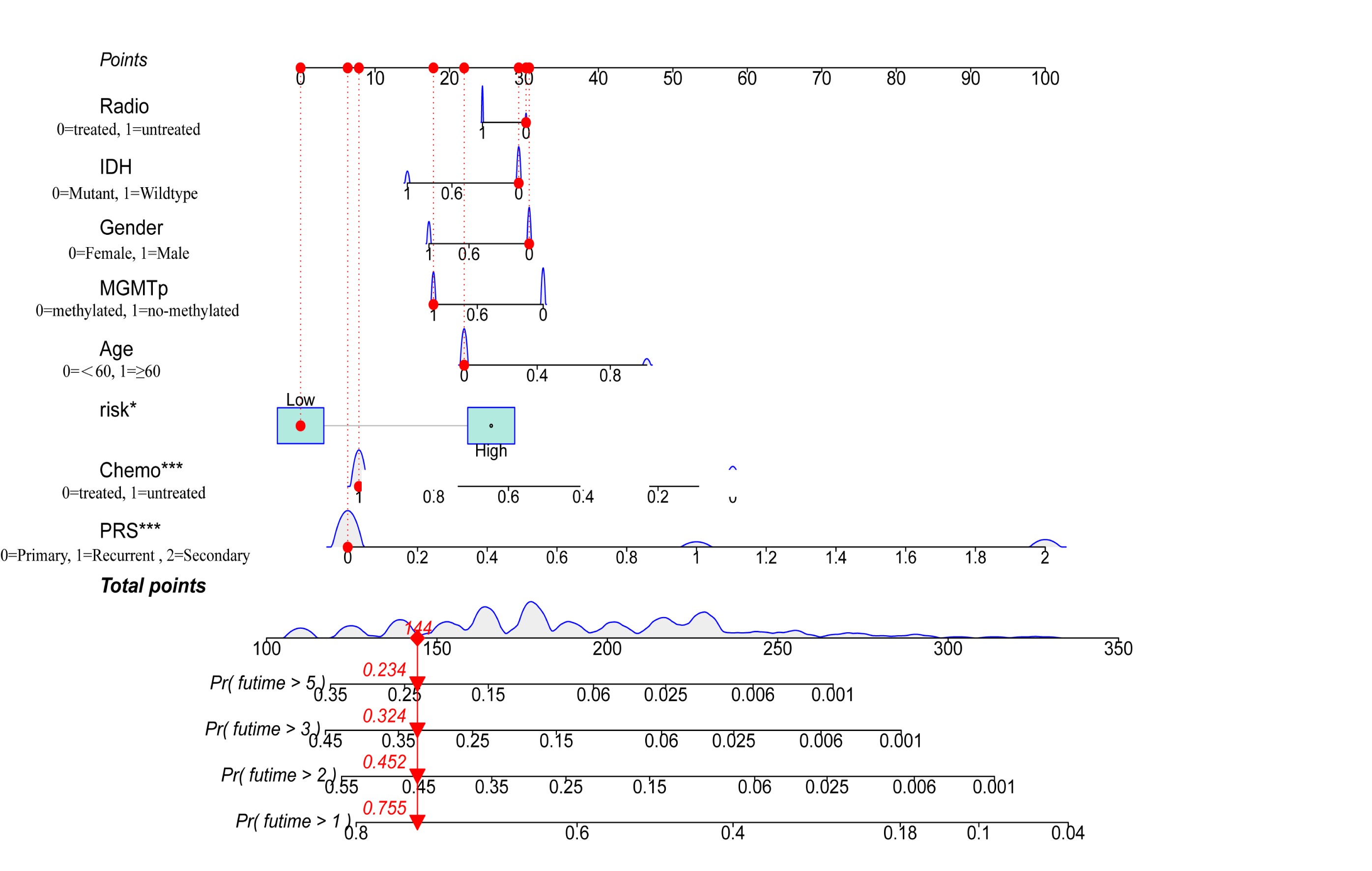

Supplement: Supplementary file 5 — Additional file 5: Figure S5. [file 12935_2022_2834_MOESM5_ESM.jpg]

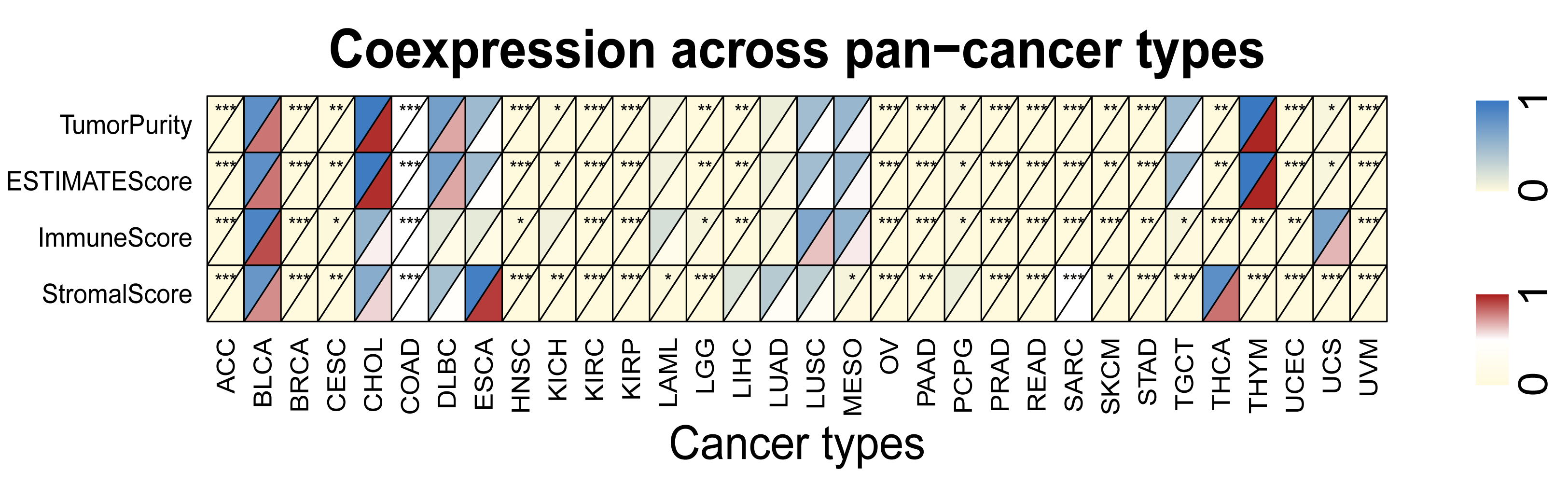

Supplement: Supplementary file 6 — Additional file 6: Figure S6. [file 12935_2022_2834_MOESM6_ESM.jpg]

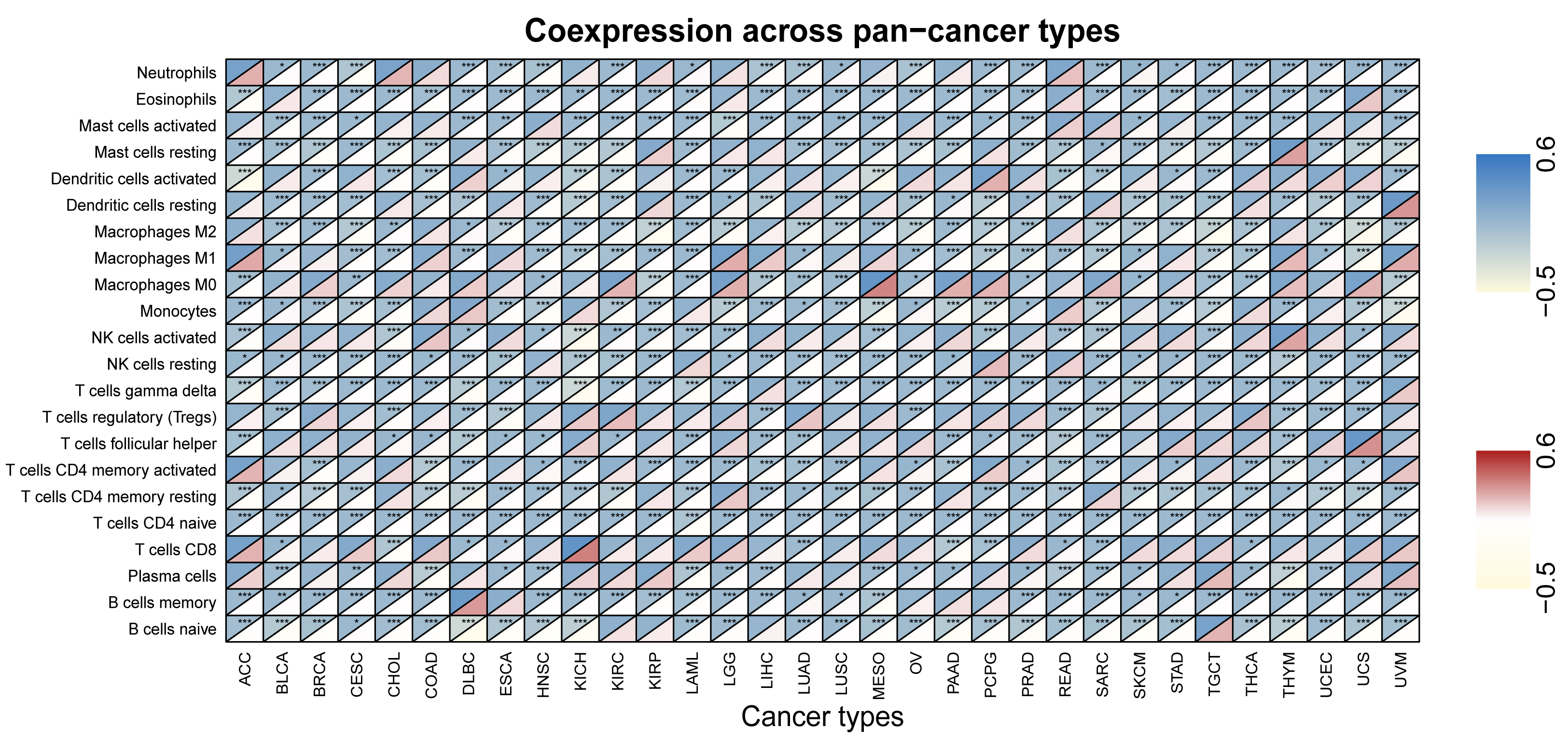

Supplement: Supplementary file 7 — Additional file 7: Figure S7. [file 12935_2022_2834_MOESM7_ESM.jpg]

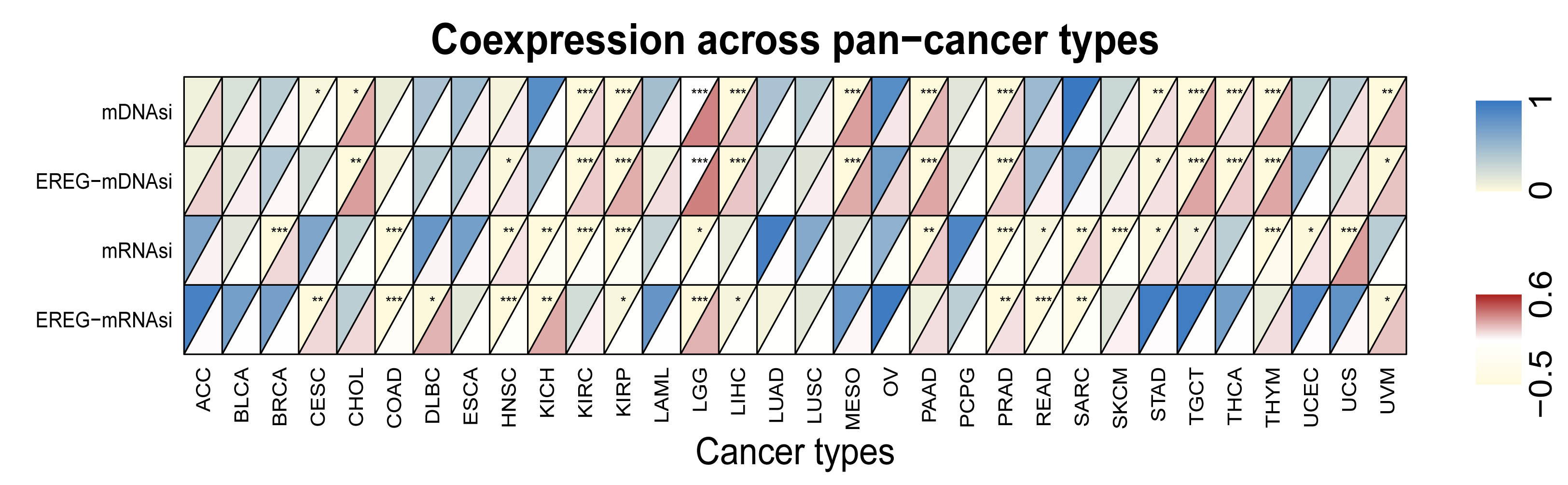

Supplement: Supplementary file 8 — Additional file 8: Figure S8. [file 12935_2022_2834_MOESM8_ESM.jpg]
